# Differential Effectiveness of Clinically-Relevant Analgesics in a Rat Model of Chemotherapy-Induced Mucositis

**DOI:** 10.1371/journal.pone.0158851

**Published:** 2016-07-27

**Authors:** Alexandra L. Whittaker, Kerry A. Lymn, Georgia L. Wallace, Gordon S. Howarth

**Affiliations:** 1 School of Animal and Veterinary Sciences, The University of Adelaide, Roseworthy Campus, Roseworthy, SA, Australia; 2 Department of Gastroenterology, Women’s and Children’s Hospital, North Adelaide, SA, Australia; Chi-Mei Medical Center, TAIWAN

## Abstract

Chemotherapy-induced intestinal mucositis is characterized by pain and a pro-inflammatory tissue response. Rat models are frequently used in mucositis disease investigations yet little is known about the presence of pain in these animals, the ability of analgesics to ameliorate the condition, or the effect that analgesic administration may have on study outcomes. This study investigated different classes of analgesics with the aim of determining their analgesic effects and impact on research outcomes of interest in a rat model of mucositis. Female DA rats were allocated to 8 groups to include saline and chemotherapy controls (n = 8). Analgesics included opioid derivatives (buprenorphine; 0.05mg/kg and tramadol 12.5mg/kg) and NSAID (carprofen; 15mg/kg) in combination with either saline or 5-Fluorouracil (5-FU; 150mg/kg). Research outcome measures included daily clinical parameters, pain score and gut histology. Myeloperoxidase assay was performed to determine gut inflammation. At the dosages employed, all agents had an analgesic effect based on behavioural pain scores. Jejunal myeloperoxidase activity was significantly reduced by buprenorphine and tramadol in comparison to 5-FU control animals (53%, p = 0.0004 and 58%, p = 0.0001). Carprofen had no ameliorating effect on myeloperoxidase levels. None of the agents reduced the histological damage caused by 5-FU administration although tramadol tended to increase villus length even when administered to healthy animals. These data provide evidence that carprofen offers potential as an analgesic in this animal model due to its pain-relieving efficacy and minimal effect on measured parameters. This study also supports further investigation into the mechanism and utility of opioid agents in the treatment of chemotherapy-induced mucositis.

## Introduction

Chemotherapy represents the first-line approach for cancer treatment, yet side-effects remain significant. One such side-effect is mucositis, which results from a series of biological events initiated by the epithelial cell response to cytotoxic damage [1]. Certain cytotoxic drugs are more commonly associated with mucositis development; the chemotherapy drug 5-fluorouracil is one such agent [2]. Mucositis affects all mucous-membrane covered surfaces from the mouth to the rectum, and remains the main dose limiting factor in cancer treatment [3, 4].

Mucositis is thought to be the resultant effect of a range of cytokine-mediated events culminating in mucosal atrophy and ulceration [5]. Epithelial sloughing, mucosal inflammation and ulceration activate nociceptors causing a direct pain response [6]. Additionally, pain may arise as a sequela of other gastrointestinal events such as abdominal bloating or a change in bowel pattern [7]. Accordingly, patients typically require potent opioid analgesics for pain control during extended periods of hospitalisation [6].

Rats are frequently used as models in alimentary mucositis disease investigations, in order to elucidate pathogenesis of the condition or to trial new therapeutics [8]. It has previously been demonstrated that rats with chemotherapy-induced mucositis undergo pathophysiological changes, [9, 10] and exhibit behavioural signs indicative of pain [11]. However, analgesic use is never reported in publications involving animal models of mucositis. It is therefore unknown if analgesics commonly used in laboratory animal practice are efficacious against the pain evoked by mucositis, or whether they impact on commonly measured experimental outcomes, and hence would confound future study interpretation.

Consequently, the current study employed a rat model of mucositis induced by 5-Fluorouracil to characterize the effect of three clinically relevant veterinary analgesics on the affective component of the pain response, gut mucosal architecture and inflammatory response. Agents chosen were: buprenorphine, a partial μ opiate agonist, [12] with moderate analgesic effect and fewer side-effects than pure μ agonists [13]; tramadol, an ‘atypical’ opioid analgesic which exerts its action via both opioid (μ receptor) and non-opioid (inhibition of monoamine uptake) mechanisms [14]; and the selective COX-2 inhibitor Non-Steroidal Anti-Inflammatory Drug (NSAID), carprofen [15].

## Materials and Methods

### Animals and Experimental Design

Female Dark Agouti rats (110-140g, n = 64) were sourced from a barrier-maintained Specific-Pathogen Free production facility (Laboratory Animal Services, the University of Adelaide, Adelaide, SA, Australia). Female rats were selected for this study since they are commonly used in mucositis disease investigations and thus results of this study would find general practical applicability [8]. On arrival, animals were group-housed in standard open-top polycarbonate rat cages of dimensions 415 mm x 260 mm x 145 mm (Tecniplast, NSW, Australia). Rats remained in these cages for an acclimatisation period of 5 days with *ad libitum* access to potable reverse osmosis treated water and a standard rat chow (Speciality Feeds, Glenn Forest, WA). Room temperature was maintained at 21–23°C with a 12 hr reversed light-dark cycle (lights off at 0800). Red light was provided to facilitate making observations from video-records in the darkness. Following acclimatisation, animals were transferred to individual housing in metabolic cages (Tecniplast. Exton, PA, US). *Ad libitum* access to food and water continued in these cages but the diet was changed to an 18% casein-based diet [9, 16] to be consistent with previously published papers in this area. [9]

Rats were randomly allocated to eight groups (n = 8): Saline injection (0.9% NaCl w/v), carprofen (15 mg/kg) + saline, buprenorphine (0.05 mg/kg) + saline, tramadol (12.5 mg/kg) + saline, 5-Fluorouracil (5-FU) injection carprofen (15 mg/kg) + 5-FU, buprenorphine (0.05mg/kg) + 5-FU, tramadol (12.5 mg/kg) + 5-FU. Experimental design was standardized with previous studies evaluating novel therapeutic agents for the treatment of chemotherapy-induced mucositis [9, 17]. All animal handling and drug administration procedures were performed as described in reputable guidance documents [18]. Rats were metabolic-cage housed on study days 0–9. Acclimatization to metabolic cages occurred on experimental days 0–2 with data recording commencing on day 3. On day 6, manually restrained animals received an intraperitoneal injection of 5-FU (150 mg/kg; Mayne Pharma Pty, Ltd, Mulgrave, Vic, Australia) or an equivalent volume of saline. At this time analgesic treatments were commenced depending on group allocation. All analgesics were injected subcutaneously into the scapular region. Analgesics were given as per clinical practice and were continued at 12 hourly intervals for the remainder of the study (a total of 6 doses). Animals were continuously video-recorded in their home metabolic cages using closed-circuit television (CCTV) cameras (OzSpy CLOC 600IR) mounted behind each cage (one camera per cage).

Body weight, food and water intake and urine output were measured daily. Rats were humanely killed by overdose of CO_2_ on day 9 (72 hours after 5-FU or saline injection). Blood was obtained via cardiac puncture and collected into lithium heparin coated blood collection tubes. All visceral organs were weighed. Gastrointestinal organs were weighed following content removal by gentle expression using a smooth edged spatula on an ice-cooled slab. Lengths of the duodenum, small intestine and colon were measured un-stretched. Sections (2 cm) of the gastrointestinal organs were collected and placed in 10% buffered formalin for histological analysis. Further (4 cm) samples were snap frozen in liquid nitrogen and stored at -80°C for later biochemical analysis. Animal housing and experimental protocols were approved by the Animal Ethics Committee of the University of Adelaide and conducted in accordance with the provisions of the Australian Code for the Care and Use of Animals for Scientific Purposes [19].

### Behavioural Data Analysis

Behavioural scoring was conducted on the continuously recorded home cage data by one person in a blinded fashion using analysis software (Cowlog, University of Helsinki, Helsinki, Finland) [20]. The occurrences of a number of behaviours previously shown to be associated with abdominal pain were scored over a 10 minute period at each of 3 time-points in the dark phase of the circadian cycle; 12 hours prior to 5-FU injection and 48 hr and 50 hours post-injection [21, 22]. The latter two time points were selected to include an observation point just prior to analgesic injection and 2 hours following injection. Frequency only, or frequency and duration, was scored based on the characteristics of the behaviour being observed. The ethogram used is illustrated in Table 1. Sampling method, behaviours scored and time points observed were selected based on a previous exploratory study in which maximal frequency of pain behaviours was demonstrated to occur at 48 hours post-chemotherapy injection [11].

**Table 1:** Ethogram for in-cage behavioural analysis (adapted from Roughan and Flecknell, 2001 [21]).

Behaviour	Description
Attend	Movement vibrissae accompanying head lifting
Back Arch	Arching of back (‘cat stretch’)
Abdominal Groom	Grooming
High Rear	Standing on hindlegs
Sleep	Inactivity for > 90s
Sniff	Movement of vibrissae
Stagger/fall	Loss of balance whilst walking or falling whilst grooming
Stop	Transient inactivity <90s
Horizontal Stretch	Elongation of the body with paws reaching above head
Turn	Turning without movement in any direction
Twitch	Contraction of back muscles
Walk	Walking
Writhe	Lateral contortion of flank muscles
Yawn	Yawning

### Myeloperoxidase (MPO) Assay

Tissue samples (4 cm) of the jejunum and ileum were thawed on ice and homogenized with 1.5 mL of phosphate buffer (10 mM, pH 6.1) for 60 seconds until a homogenous solution was obtained. Homogenized samples were kept at −80°C until required. MPO levels were determined using the assay described by Krawisz *et al*. *1984* [23]. Homogenates were thawed on ice and centrifuged at 13000 g for 13 min. Following removal of the supernatant the cell pellets were re-suspended in hexadecyltrimethyl ammonium bromide (0.5%, pH 6.0). Samples were then vortexed for 2 min and centrifuged at 13000 g for 3 min. Supernatants were collected into a 96- well plate and reacted with *o*-dianisidine. Absorbance was measured at 450 nm at 1 min intervals for a period of 15 min using a microplate reader (Sunrise Microplate Reader, Tecan Austria GmbH, Grodig, Austria). A pre-designed excel macro was used to calculate MPO activity. Activity was expressed as units MPO per gram of tissue.

### Blood Biochemistry

Blood samples were centrifuged at 4000 *g* for 6 min, the plasma supernatant removed and immediately analyzed for biochemical parameters using Beckman Coulter reagents (Beckman Coulter AU480, NSW, Australia) at a commercial diagnostic lab (Veterinary Diagnostics Laboratory, The University of Adelaide, Australia). Analyses included albumin, alkaline phosphatase (ALP), alanine transaminase (ALT), amylase, Ca:P ratio, creatinine kinase, creatinine, glucose, Na:K ratio, total protein and urea.

### Histological Analysis

Tissue samples were fixed in formalin for 24 h and then placed in 70% ethanol. Transverse tissue sections (4 μm) were embedded in paraffin wax, H&E stained and viewed using a light microscope (Olympus CX-41; Olympus, Tokyo, Japan). The disease severity score was determined semi-quantitatively by scoring eight independent histological criteria. The method used represented a slight modification of that described by Howarth *et al*. 1996 [24]. The criteria scored were: villus blunting, crypt distortion, reduction in goblet cell number, dilation of lymphatics, thickening of the submucosa, thickening of the muscularis externa, enterocyte disruption and lymphocytic cell infiltration. Each criterion was scored from zero (normal) to three (maximal damage) and expressed as a median score. Saline control rat intestinal tissue was used as a baseline reference to grade the criteria. Villus heights and crypt depths (40 villi and 40 crypts per section) were determined in the jejunal and ileal sections using a light microscope (Nikon, ProgRes®CS, Tokyo, Japan) and image ProPlus software version 5.1 (Media Cybernetics, Silver Spring MD, USA). All histological analyses were conducted in a blinded fashion.

### Statistical Analyses

Statistical analyses were conducted using PASW 21 (SPSS, Inc., Chicago, IL, USA) and Megastat Excel Add-In (version 10.2, McGraw-Hill Higher Education, New York, NY). Data were tested for normality and homogeneity of variance using the Shapiro–Wilk test. Parametric data were compared using one-way analysis of variance (ANOVA) with a Tukey's *post-hoc* test and presented as means ± standard error of the mean. Disease severity score and serum biochemistry were analysed by a Kruskal-Wallis test with a Mann Whitney U-test to determine between-group significance. Rat behavioural data were also non-parametric and analyzed similarly with both between, and within group comparisons made. Non-parametric data are shown as medians and ranges. Significance was determined at *p* < 0.05 with Bonferroni corrections made where appropriate to account for multiple comparisons.

## Results

### Daily Metabolic Parameters and Bodyweight

Following saline or 5-FU injection there were significant differences between the groups in a number of the clinical parameters recorded (Table 2; see also S1 File). In the analgesic control groups receiving saline injection, both buprenorphine and tramadol resulted in a decrease in bodyweight with no concomitant decrease in food consumption. Bodyweights of animals in the remaining treatment groups demonstrated an increase. Buprenorphine administration increased water intake and consequently urine output (Table 2).

**Table 2:** Effects of analgesic agents on cumulative bodyweight change, food and water intake and urine output in saline-injected rats and in 5-FU injected rats from days 7–9. Data are expressed as means ± SEM.

	saline alone	saline + carprofen	saline + buprenorphine	saline + tramadol	5-FU alone	5-FU + carprofen	5-FU + buprenorphine	5-FU +tramadol
Body Weight Change (%)^§^	3.4 ± 1.1	3.6 ± 0.5	-0.3 ± 0.9*	-1.4 ± 0.3*	-4.2 ± 0.6*	-5.9 ± 0.4*	-7.2 ± 0.8†*	-10.2 ± 0.5†*
Mean Daily Water Intake (ml/gbw)	0.18 ± 0.02	0.19 ± 0.04	0.43 ± 0.10*	0.26 ± 0.06	0.23 ± 0.03	0.29 ± 0.03	0.54 ± 0.10*†	0.50 ± 0.07*†
Mean Daily Food Intake (g/gbw)	0.08 ± 0.01	0.06 ± 0.01	0.06 ± 0.01*	0.06 ± 0.01	0.03 ± 0.01*	0.03 ± 0.01*	0.03 ± 0.01*	0.02 ± 0.01*
Mean Daily Urine Output (ml/gbw)	0.09 ± 0.01	0.20 ± 0.05*	0.21 ± 0.05*	0.10 ± 0.01	0.16 ± 0.02	0.16 ± 0.02	0.34 ± 0.07*†	0.49 ± 0.05*†

* indicates statistical significance compared to saline at *p* < 0.05.

† indicates statistical significance compared to 5-FU alone at *p* < 0.05.

§ Bodyweight change is expressed as the mean total % change calculated in relation to the baseline weight recorded at the time of saline/5-FU injection.

Administration of 5-FU significantly reduced bodyweight and food intake compared to saline injected rats (Table 2). In the rats administered either buprenorphine or tramadol in combination with 5-FU the analgesic agents potentiated this weight loss compared to the 5-FU control group (↑72%, *p* = 0.005; ↑143%, *p* < 0.001 respectively). Again, notably, this bodyweight decrease was not accompanied by a significant decrease in food intake (*p =* 0.99 for both analgesic agents). Buprenorphine and tramadol administration to 5-FU injected rats increased water consumption in comparison to 5-FU control animals by 135% (*p* = 0.002) and 117% (*p* = 0.003) respectively. Correspondingly, urine output was increased in animals receiving these analgesics.

### Behavioural Assessment Scoring

In general, the specific pain behaviours of interest were observed at low frequencies, hence the behaviours that had previously been shown to increase in mucositis [11] were amalgamated to form a composite pain score, as described previously [25, 26]. This score consisted of back arch, horizontal stretch, stagger, twitch and writhe. Whilst it is known that metabolic cage housing does evoke changes in animal behaviour [27, 28] the consistent use of these cages across all treatment groups should have minimized any confounding influence of the cages themselves on behavioural outcomes.

**Fig 1:**
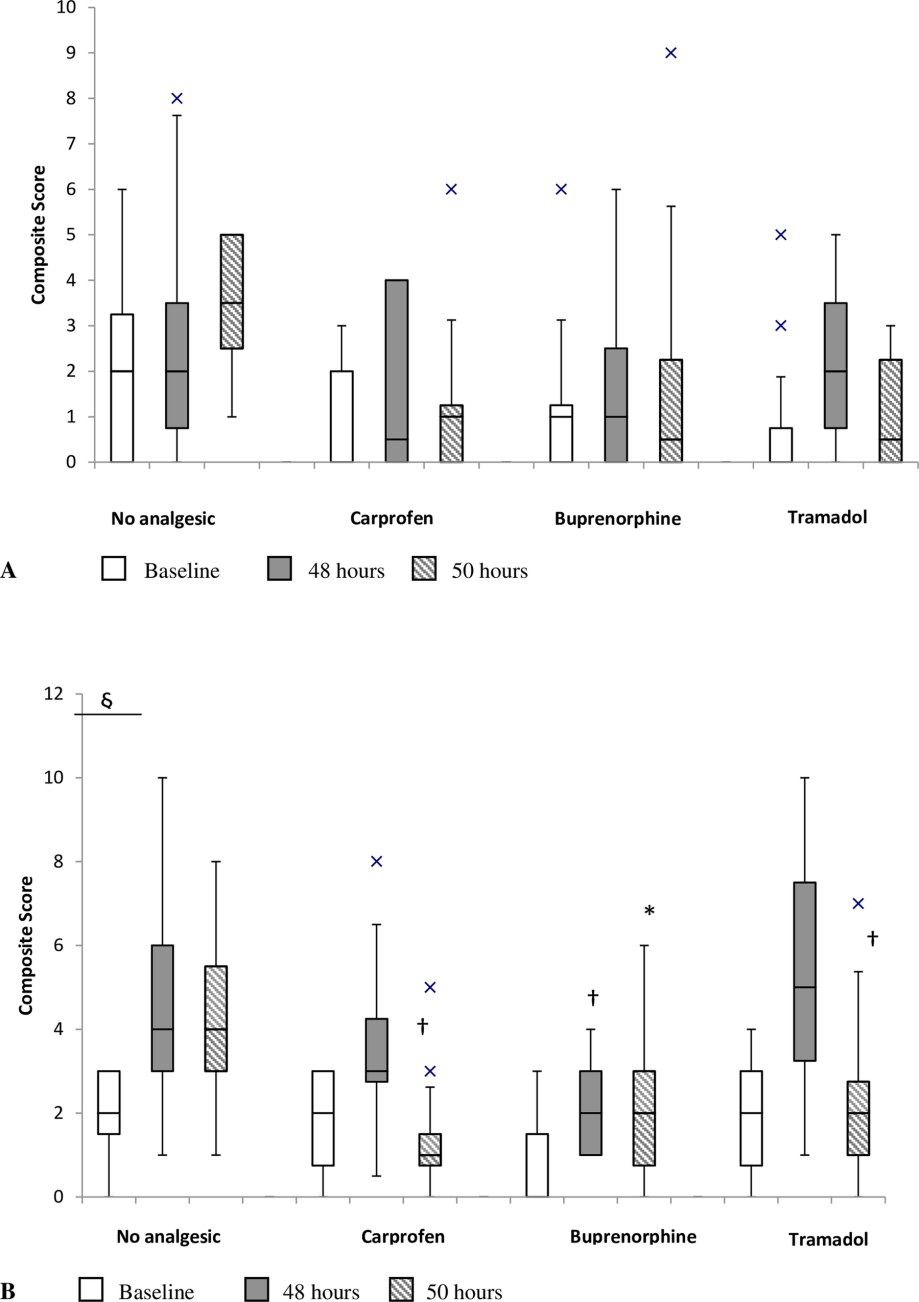
Effect of analgesic agents on composite pain score prior to injection, and 48hr and 50 hours after injection; (A) saline injection (B) 5-FU injection. The box plots represent the first and third quartiles, horizontal lines represent the median pain score and the whisker ends represent 1.5 x inter-quartile range, X represent outlying points. * indicates *p* < 0.05 compared to saline + no analgesic and † *p* < 0.05 compared to 5-FU + no analgesic between groups within the same time point. § represents significant difference at p = 0.008 between time points within the same group.

Whilst all analgesic agent-treated groups demonstrated significant decreases in score when compared to animals that received 5-FU alone at either the 48 hour or 50 hour time point, with the exception of buprenorphine (50 hours) these values were not significant in comparison to saline controls alone (Fig 1; see also S2 File). However, on comparing scores within groups between the time points a significant difference was only recorded with the 5-FU control animals between baseline and 48 hours (*p* = 0.01) indicating that in all other groups they either experienced no change in pain level, or that pain was effectively treated.

There was less consistency in the frequency of performance of the other measured behaviours (Table 3; see also S2 File) and there was significant inter-animal variability. Key changes included that high rearing increased between baseline and 50 hours in the 5-FU alone group (*p* = 0.03) and sleep duration increased between baseline and 48 hours in the buprenorphine treated animals with mucositis (*p* = 0.01). The converse was observed for tramadol treated animals (*p* = 0.03).

**Table 3:** Effect of analgesic agents on non-pain specific behaviours. Data are expressed as median (interquartile range) of behavioural frequency over 10 minute observation period unless duration indicated.

	saline alone	saline + carprofen	saline + buprenorphine	saline + tramadol	5-FU alone	5-FU + carprofen	5-FU + buprenorphine	5-FU +tramadol
***Baseline***								
High rear	0 (2)	0 (0)	1 (3.5)	0 (0)	0 (0)§ ^(base, 50)^	0 (0)*	0 (2)	1(0.75)
Abdominal groom	2.5 (4)	1 (0.5)	1 (1)	0 (0)	0.5 (2.5)	0 (0)	2.5 (2.5)	1 (0.75)
Stop	2 (3.5)	1 (1.5)	2 (3.5)	0 (3)	3.5 (2.25)	0 (0.5)†	3 (3.75)	6 (1.5)*
Sleep Duration (s)	364 (563)	516 (237)	149 (295)	600 (185)	377 (230)	490 (179)	79 (247)	290 (219)
***48 Hours***								
High rear	0 (0)	0 (0)§ ^(48,50)^	0 (1)	0 (2.5)	0 (0.5)	0 (0)	0 (0)	0 (0)
Abdominal groom	1 (2)	1 (1)	0 (2)	1 (1.5)	0.1 (2)	1.5 (3)	0 (0.5)	1 (1)
Stop	0.5 (4.5)	0 (1.5)	1 (3.5)	5 (5.5)*§ ^(base, 48)^	4 (2)	4.5 (3.25)§^(base, 48)^	1 (0.75)†	4.5 (5.25)
Sleep Duration	402 (333)	478 (129)	582 (597)	0 (194)*	402 (143)	177 (440)	543 (185)§ ^(base, 48)^	0 (0)†§^(base, 48)^
***50 Hours***								
High rear	1 (2)	1 (2.5)	0 (0)	0 (0)	1.5 (5.5)	0 (0.5)	0 (0.5)	0.5 (2.5)
Abdominal groom	2 (0.75)	2 (4)	0 (0)	1 (2)	1 (1.75)	1 (1.5)	0 (0)*†	0.5 (1.5)
Stop	3 (2.25)	2 (2.5)	1 (2.5)	4 (5)	5.5 (3.75)	1.5 (2.5)	3 (3)	2.5 (5.25)
Sleep Duration	46 (292)	157 (302)	534 (109)	415 (433)	98 (94)	366 (183)	337 (252)	63.5 (177)

* indicates statistical significance at *p* < 0.05 compared to saline alone.

† indicates statistical significance at *p* < 0.05 compared to 5-FU alone.

§ represents significant difference between time points within the same group, superscript value shows the time points that are statistically different.

### Organ Weights

In healthy analgesic control animals, carprofen increased liver weight by 12% in comparison to saline alone control animals (*p* = 0.04). Alternately, buprenorphine decreased liver weight by 10% (*p* = 0.03, Table 4). Administration of 5-FU significantly reduced thymus weight by 60% (*p* < 0.001) and spleen weight by 20% (*p* < 0.001) compared to saline-injected rats (Table 4). Both buprenorphine and tramadol administration exacerbated the 5-FU induced decrease in thymic weight. Carprofen elevated renal weights (~↑20%, p ≤ 0.001) whilst the converse was observed with tramadol (↓12%, *p* = 0.01) compared to 5-FU control (Table 4; see also S1 File). Administration of 5-FU had no effect on liver mass. Analogously, carprofen led to an increase in liver weight (11%, *p* = 0.006) in comparison to 5-FU control animals, whilst tramadol decreased liver weight (11%, p = 0.02). However, buprenorphine when co-administered with 5-FU failed to produce liver mass changes.

**Table 4:** Effects of analgesic agents on visceral organ weights of female Dark Agouti rats 72 hr after 5-FU or saline injection. Organ weights were calculated as % (wtg/g/bwt) and are expressed as mean % ± SEM. All values are x 10^−2^.

	saline alone	saline + carprofen	saline + buprenorphine	saline + tramadol	5-FU alone	5-FU + carprofen	5-FU + buprenorphine	5-FU +tramadol
Thymus	20 ± 1	20 ± 2	17± 1	20 ± 1	8 ± 1*	8 ± 1*	5 ± 1*†	6 ± 1*†
Liver	393 ± 12	440 ± 9*	357 ± 6*	389 ± 22	388 ± 1	429 ± 9*†	381 ± 9	347 ± 9*†
Spleen	21 ± 1	23 ± 1*	23 ± 1	21 ± 1	17 ± 1*	18 ± 1*	15 ± 1*	16 ± 1*
Left Kidney	41 ± 1	47 ± 1*	42 ± 1	41 ± 2	44 ± 1	50 ± 1*†	47 ± 2*	39 ± 2†
Right Kidney	43 ± 1	48 ± 1*	43 ± 1	42 ± 2	45 ± 1	50 ± 1*†	49 ± 1*	40 ± 1†
Adrenal Glands	2 ± 0.1	2 ± 0.3	2 ± 0.1	2 ± 0.1	2 ± 0.1	3 ± 0.1	3 ± 0.1	3 ± 0.1

* indicates statistical significance at *p* < 0.05 compared to saline alone.

† indicates statistical significance at *p* < 0.05 compared to 5-FU alone.

5-FU significantly reduced the gastrointestinal organ lengths such that a 9% reduction was evident in the jejuno-ileum (*p* = 0.01) and an 11% reduction in the colon (*p* = 0.03) (Table 5; see also S1 File). The analgesic agents had minimal moderating effect on organ lengths except for tramadol which attenuated the reduction in colon length produced by 5-FU (*p* = 0.02). However, all analgesic agents produced a reduction in weight of the jejuno-ileum when compared to 5-FU controls. A similar effect was observed in colon weights although this did not attain significance in the 5-FU + carprofen group (*p* = 0.08).

**Table 5:** Effects of analgesic agents on gastrointestinal organ weights and lengths of female Dark Agouti rats 72 hr after 5-FU or saline injection. Organ weights were calculated as % (wtg/g/bwt) and are expressed as mean % ± SEM. Lengths were measured in cm and are presented as mean ± SEM.

	saline alone	saline + carprofen	saline + buprenorphine	saline + tramadol	5-FU alone	5-FU + carprofen	5-FU + buprenorphine	5-FU +tramadol
**jejenum + ileum**								
**Weight (x10**^**-2**^**)**	300 ± 10	246 ± 6*	224 ± 10*	229 ± 10*	250 ± 6*	223 ± 3*†	200 ± 4*†	200 ± 8*†
**length**	70.5 ± 1.8	72.1 ± 0.5	73.3 ± 0.7	74.6 ± 1.7	64.8 ± 2.3*	61.3 ± 0.9*	63 ± 1.9*	66.8 ± 1.8
**colon**								
**Weight(x10**^**-2**^**)**	70 ± 10	64 ± 2*	65 ± 3	63 ± 5*	70 ± 3	67 ± 2	62 ± 3*†	60 ± 3*†
**length**	14.4 ± 0.7	14.3 ± 0.4	14.9 ± 0.5	15.1 ± 0.6	12.8 ± 0.6*	11.0 ± 0.2*	12.7 ± 0.5*	14.4 ± 0.4†

* indicates statistical significance at *p* < 0.05 compared to saline alone.

† indicates statistical significance at *p* < 0.05 compared to 5-FU alone.

### Serum Biochemistry

Serum biochemistry results are reported in Table 6. Not all biochemical parameters were available for the tramadol-treated groups. When compared with saline controls, 5-FU caused statistically significant differences in creatinine kinase (CK) and creatinine values. However, these changes were unlikely to be of clinical significance. Noteworthy changes included; the elevation in creatinine in all 5-FU treated groups, and the depression in total protein in animals receiving the combination of 5-FU and carprofen. The consistent depression in urea was likely to be attributable to the specific analyzer used. It should be noted that serum biochemistry reference ranges for Dark Agouti rats were not available.

**Table 6:** Effects of analgesic agents on blood biochemistry values of female Dark Agouti rats 72 hr after 5-FU or saline injection. Data are expressed as median values with experimental group range in brackets. Shaded results are outside references range values given by Giknis and Clifford, 2008 (Charles River labs for inbred Wistars).

	saline alone	saline + carprofen	saline + buprenorphine	saline + tramadol	5-FU alone	5-FU + carprofen	5-FU + buprenorphine	5-FU + tramadol	Ref Interval
Alkaline phosphatase (U/L)	77.0 (32–135)	114.0 (99–135) †	99.5 (85–115)	-	60.5 (42–139)	63.0(60–85)	64.5 (57–71)	-	26–147
Alanine Transaminase(U/L)	42.0 (24–46)	51.0 (38–61) †	46.0 (31–69) †	-	30.0 (18–42)	29.0 (21–40)	40.5 (33–56)	-	16–48
Amylase (U/L)	399.0 (380–546)	380.0 (316–1040)	385.0 (295–399) †	-	553.0 (371–645)	509.0 (446–776)	451.0 (389–550)	-	NA
Ca:P ratio	0.8 (0.6–1.1)	0.8 (0.7–0.8)	0.7 (0.5–0.9)	-	0.9 (0.5–1.2)	0.8 (0.7–0.8)	0.7 (0.4–0.8)	-	NA
Creatinine Kinase (U/L)	631 (255–1776)	304 (188–599)	428 (120–700)	-	300 (98–1182)*	444 (230–2019)	346 (165–1222)	-	163–1085
Creatinine (μmol/L)	21.9 (18–27)	21.5 (19–24)	27.5 (22–39)	26.0 (21–31)	28.0 (20.6–33)*	26.0 (23–31)*	34.5 (28–45) *†	32.2 (28–41) *†	11–33
Glucose (mmol/L)	7.0 (5–10)	10 (7–13)	12.0 (7–13)*†	-	7.3 (3–10)	9.5(7–12)	9.5 (6–16) †	-	4.2–9.7
Na:K ratio	22.4 (18–26)	20.8 (20–29)	20.4 (18–23) †	-	24.6 (20–30)	25.9 (20–29)	24.3 (20–25)	-	NA
Total Protein (g/L)	58.0 (55–64)	51.5 (50–56) *†	59.0 (55–62)	-	59.0 (52–63)	48.5 (43–57) *†	56.5 (42–66)	-	55–77
Urea (mmol/L)	5.0 (4–6)	5.0 (4–5)	5.5 (5–9)	6.0 (5–7)	4.3 (3–7)	5.0 (4–6)	4.1 (4–6) *	7.3 (5–8) *†	13.2–27.1

* indicates statistical significance at *p* < 0.05 compared to saline alone.

† indicates statistical significance at *p* < 0.05 compared to 5-FU alone.

### Myeloperoxidase Activity

There was no significant difference in MPO activity in either the jejunum or ileum when analgesics were administered to healthy control animals, indicating that the agents caused no adverse effects on inflammation at the given dosages.

**Fig 2:**
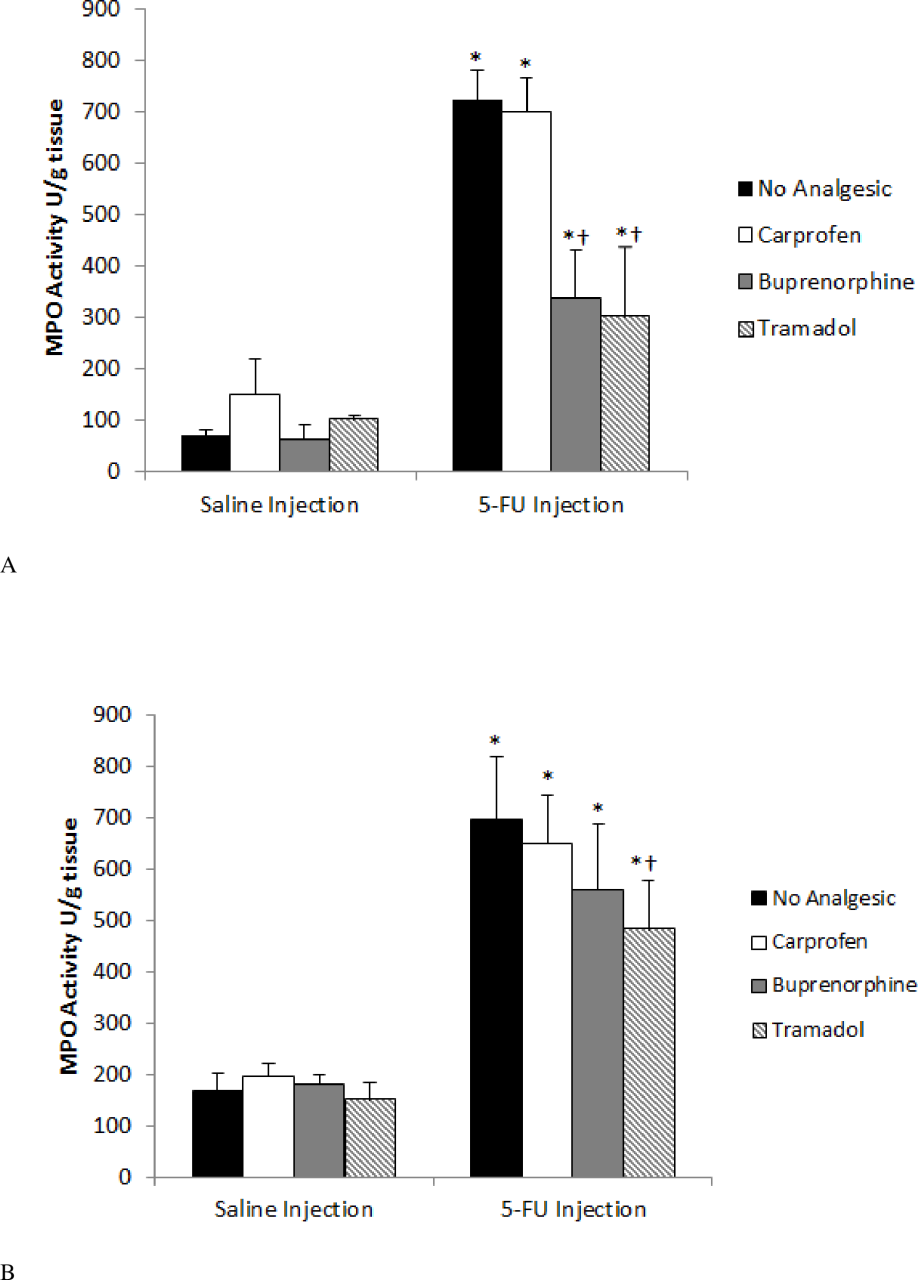
Effect of analgesic agents on myeloperoxidase (MPO) activity in the jejunum (A) and ileum (B) 72 hr after either saline or 5-FU injection. Data are expressed as mean (MPO units/g tissue) ± SEM. * indicates *p* < 0.05 compared to saline + no analgesic and † *p* < 0.05 compared to 5-FU + no analgesic.

Injection of 5-FU caused a significant (*p* < 0.001) increase in MPO activity in the proximal jejunum and ileum of 5-FU control (1046% and 412% respectively) compared to saline control animals (Fig 2; see also S3 File). In the jejunum, both buprenorphine and tramadol caused a highly significant decrease in MPO activity in comparison with chemotherapy-treated control animals (53%, p = 0.0004 and 58%, p = 0.0001 respectively, Fig 2A). Analogously, in the ileum, tramadol reduced MPO activity by 31% (*p* = 0.003) in comparison to 5-FU controls. However, buprenorphine failed to have a similar ameliorating effect (*p* = 0.19, Fig 2B).

### Histological Severity Score

**Fig 3:**
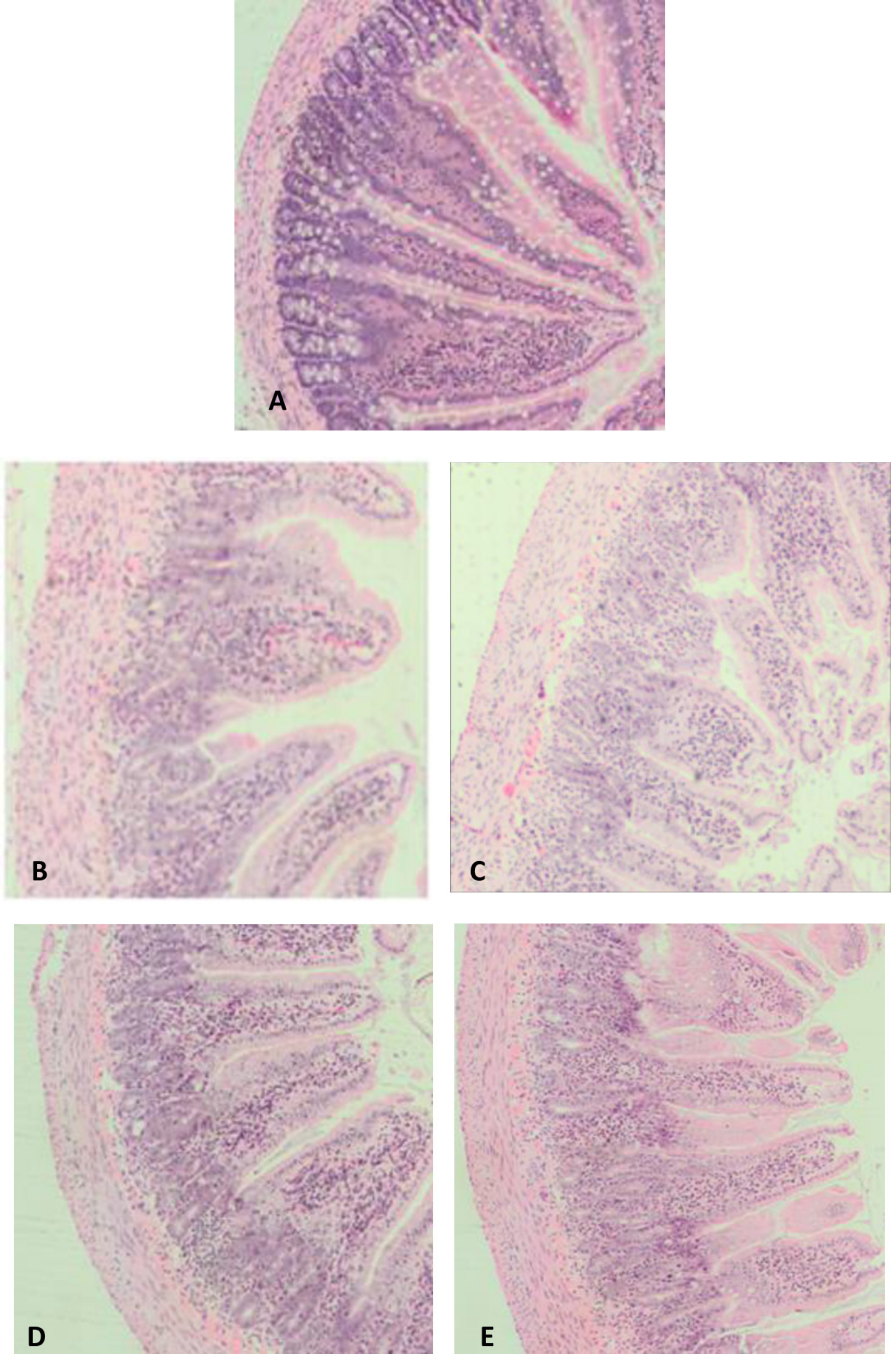
Representative photomicrographs (x 100) of the proximal jejunum sections stained with haematoxylin and eosin in animals treated with saline alone (A), 5-FU alone (B), 5-FU + carprofen (C), 5-FU + buprenorphine (D), 5-FU + tramadol (E).

**Fig 4:**
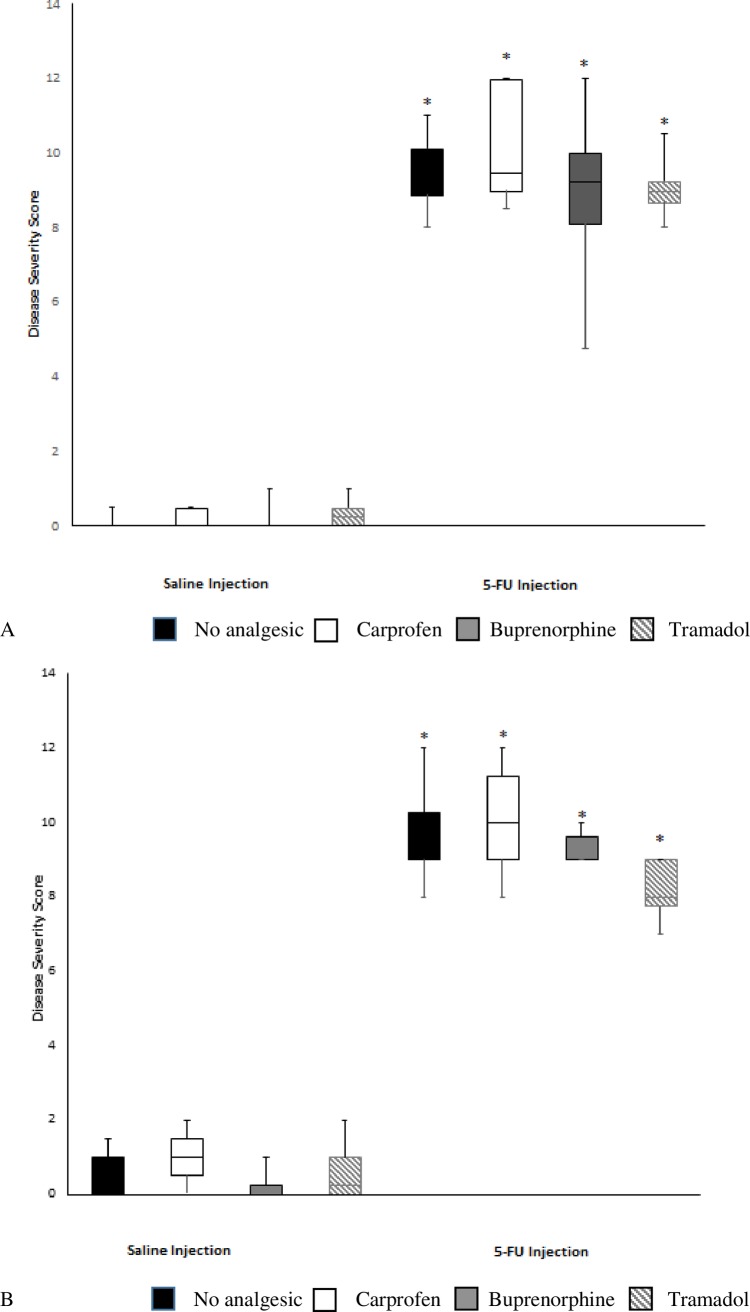
Effects of analgesic agents on disease severity score in the jejunum (A) and ileum (B) 72 hr after either saline or 5-FU injection. The severity scores were rated based on 8 parameters on different layers of intestinal tissues. The box plots represent the first and third quartiles, horizontal lines represent the median disease severity score and the whisker ends represent the maximum and minimum score. * indicates *p* < 0.05 compared to saline + no analgesic.

The histological architecture of the jejunum and ileum was entirely consistent with previous rat studies of mucositis, and included shortening of villi, crypt disruption, loss of goblet cells and enterocyte disruption (Fig 3). Chemotherapy administration significantly increased disease severity score in the proximal jejunum and ileum in all treated animals (Fig 4; see also S4 File) in comparison with saline-injected control animals (*p* < 0.001 for all), thus determining that the mucositis condition was present. The analgesic agents had no effect on histological severity score in healthy animals or when given in conjunction with 5-FU (Fig 4).

### Villus Height and Crypt Depth Measurements

#### Jejunal Effects

**Fig 5:**
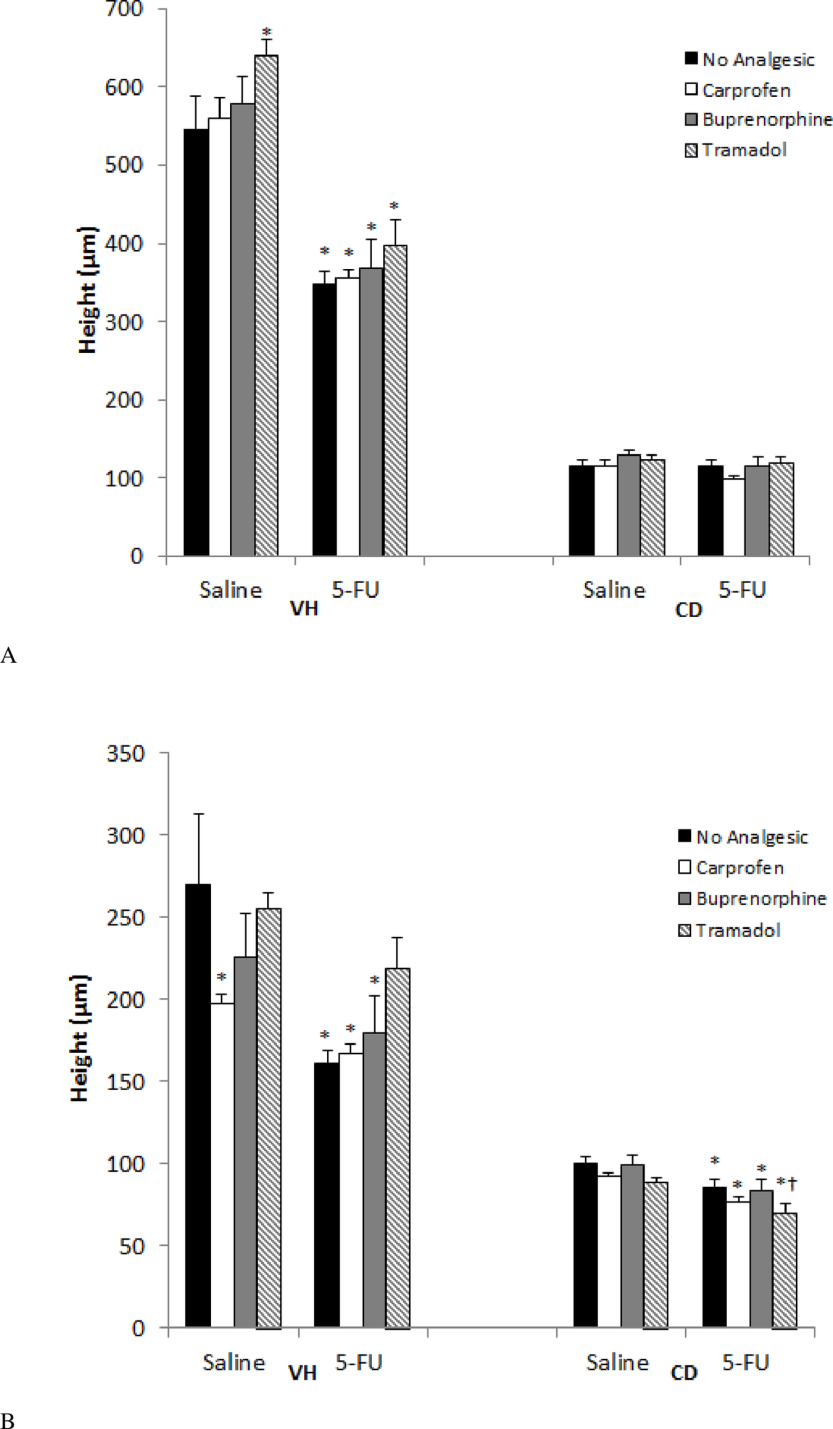
Effects of analgesic agents on villus height and crypt depth in the jejunum (A) and ileum (B) 72 hr after either saline or 5-FU injection. VH = villus height, CD = crypt depth. Data are expressed as mean ± SEM. * indicates *p* < 0.05 compared to saline + no analgesic and † *p* < 0.05 compared to 5-FU + no analgesic.

5-FU injection caused a shortening of the villi in the jejunum (36%, *P*<0.001) compared to no analgesic saline controls (Fig 5; see also S4 File). Tramadol had a lengthening effect on villus height when administered with 5-FU, although this failed to attain significance. Administration of 5-FU caused no general reduction in crypt depth.

#### Ileal Effects

Similarly, 5-FU caused a villus shortening in the ileum (40%, *p* = 0.005) compared to no analgesic saline controls (Fig 5B). Tramadol, when administered in the presence of 5-FU had a lengthening effect on villus height. This lengthening normalized villus heights towards values in saline treated (219 ± 18 v 270 ± 43 μm, respectively, *p* = 0.18, Fig 5B). However, it should be noted that the 5-FU + tramadol villus height values were not significant with respect to the 5-FU control group (*p* = 0.12, Fig 5B). Contrary to the results observed for jejunal tissue, 5-FU caused a general reduction in crypt depth when compared with that of saline control animals.

## Discussion

This study represents the first published investigation into analgesics administered to rats with experimentally-induced mucositis. The non-use of analgesia in these models potentially poses an ethical issue. However, an ethical issue also arises if an administered analgesic confounds data interpretation, resulting in misleading results and unnecessary study repetition. The current study indicated that all analgesics tested were efficacious against rat nociceptive behaviour. However, the opioid agonists had the potential to confound interpretation of study results due to an anti-inflammatory action. Therefore, in mucositis studies in which myeloperoxidase activity and histological parameters are the research outcomes of interest, carprofen is the preferred analgesic refinement.

Indeed, the opioid agents had more striking effects on the daily metabolic parameters and bodyweight than the NSAID. Importantly, bodyweight decreased after administration of either buprenorphine or tramadol in healthy animals, and these agents potentiated weight loss in animals with mucositis. This finding is likely to have arisen due to appetite suppression as a result of excess opioid activity, [29] postulated to be due to increased sedation or a nauseating effect. However, we failed to consistently demonstrate a similar decrease in food consumption in animals treated with these agents. The increase in drinking behaviour was expected since it is well established that μ-receptor activation leads to increased water intake, [30] although activation of this receptor also tends to lead to urinary retention, [31] rather than an increase in urination as demonstrated in the current study. The partial selectivity for this receptor by buprenorphine, and low receptor affinity by tramadol [32] therefore present significant clinical advantages of these drugs.

The analysis of occurrence of specific pain behaviours in rats is a well-validated pain assessment technique [21, 25, 33]. However, to date the applicability of the technique to models of chemotherapy-induced mucositis has only been reported once [11]. In the current study, intra-group differences across time were only evident in the 5-FU alone treated animals. There are two explanations for this finding; either 1) no pain was experienced by animals in all other groups and hence no change in score yielded, or 2) pain was experienced by animals with mucositis and was effectively managed at these time-points, hence no change in score was recorded. The latter interpretation is preferred for the 5-FU treated groups since the daily metabolic parameters and bodyweight data suggested that pathophysiological processes associated with mucositis development were occurring. The behavioural data provide evidence for the analgesics being present in plasma at a pharmacologically active level. This would however require confirmatory pharmacokinetic determination. Furthermore, by this interpretation it is concluded that, as previously determined, [21] the behaviours were truly pain-specific and not modified by drug-induced states such as sedation. Nevertheless, these conclusions are advanced cautiously since inter-group comparisons did not yield statistically significant differences between the chemotherapy-treated groups and saline control animals. However, given the inherent inter-individual variability in behaviour performance, and the subjective nature of the pain experience, comparison of values for the same animals across time may yield more accurate and relevant results. Use of less manually intensive automatic home-cage monitoring in future studies would allow comparison of a greater number of individuals at more time points to confirm these conclusions [34].

There is a significant body of literature demonstrating that opioids modulate immune responses [35]. Chronic administration of opioids to rodents has been demonstrated to lead to thymic and splenic atrophy, [36–38] and elevation of CD4+/CD8+ ratios, [38] yet other studies in rats have demonstrated an increase in spleen weight [39]. Such effects may be brought about through direct suppression of natural killer cell activity via opiate receptors, or indirectly through modulating cytokine release, [40] or induction of corticosteroids [41]. However, buprenorphine has minimal immunosuppressive action [42, 43]. Since the splenic weight decrease reported in the current study only occurred when co-administered with 5-FU there may have been a synergistic effect, possibly driven via the chemotherapy-induced inflammatory response, and the consequent Hypothalamic Pituitary Axis induced increase in corticosterone, [44] or as a direct immunosuppressive effect of 5-FU. Consistent with our results, opioids have previously been shown to decrease liver weights and increase kidney weights *in vivo* [39]. A decrease in hepatic vacuolization due to reduced food intake and hence glycogen storage may explain the former finding [39]. The increase in renal and hepatic weight in carprofen-treated animals is postulated to be a result of the drug clearance activities of these organs. It is noteworthy that despite the previously referred to changes in organ weights, serum biochemistry confirmed there were no organ functional changes. Opioids tend to contract intestinal muscle via direct activation of muscle cells, [45] or depression of nitric oxide release from enteric neurons [46]. The finding of an increased colon length in the tramadol group is therefore inconsistent but may reflect the differential selectivity of buprenorphine and tramadol for the μ-opioid receptor.

Myeloperoxidase enzyme levels provide a measure of neutrophil influx and hence acute inflammation [23]. Administration of the opioid analgesics had an anti-inflammatory effect as evidenced through the substantial reduction in myeloperoxidase activity. This effect is assumed to be as a result of the μ-receptor agonism since a similar effect has been noted previously [47]. This is of interest since opioid receptor agonists are generally assumed to have poor, or at least misunderstood anti-inflammatory action [48]. To our knowledge, this is the first report of this finding in chemotherapy-induced mucositis. In order to confirm this mechanism and determine the magnitude of it future studies should incorporate a μ- receptor antagonist agent.

Interestingly, the NSAID carprofen failed to attenuate the MPO response. This attests to the selectivity of NSAID agents in preventing the production of inflammatory mediators such as prostaglandins and leukotrienes via inhibition of the cyclooxygenase pathway [49]. However, phagocytosing polymorphonuclear leucocytes produce H_2_O_2_ and chloride. The interaction between H_2_O_2_ and chloride ions with MPO releases oxidant products. These oxidant products are likely to be important inflammatory mediators [49]. Therefore, in spite of the blockade of cyclooxygenase by NSAIDs, an inflammatory effect could still be produced via this alternate oxidant pathway [49]. Therefore the ideal NSAID would not only have effects on the cyclooxygenase pathway, but anti-oxidant/free radical scavenging ability in addition. Whilst it has been demonstrated that a range of NSAIDS target polymorphonuclear MPO via their free radical scavenging effects, [49] our data suggest that carprofen was unable to exert this effect. Since, myeloperoxidase activity is commonly used to measure therapeutic effect in trials of new therapeutic agents against mucositis, carprofen is rendered the drug of choice by this finding.

Despite, the beneficial reduction in MPO activity caused by the opioid agents, in the current study, there was no corresponding decrease in histological severity score. However, tramadol as the most efficacious agent in reducing MPO, showed a trend towards restoration of villus height. It is noteworthy that this effect also occurred in healthy animals and is previously unreported. In order to fully understand the inflammatory processes occurring in chemotherapy-induced mucositis, their relation to histological repair and synergism between therapeutics, future studies should investigate a range of inflammatory markers such as the pro-inflammatory cytokines and cyclooxygenase activity.

Mechanisms of pain production by cytotoxic agents in gastrointestinal mucositis are poorly understood, [50] yet pain remains one of the key dose-limiting factors for patients [51]. However, research effort in this area is likely to increase since the discovery that: 1) chemotherapy-related hyperalgesia can be reversed by immunomodulatory agents [51]; and 2) there is a high level of μ-opioid receptor expression in the intestinal tract, particularly during inflammation [47]. Therefore, the ability to reliably measure affective pain as opposed to nociception is a much needed animal model refinement. This study has indicated that behavioural pain assessment scoring may be one such tool but does require further validation. Our data do however raise some concerns with the ability of the model to replicate the human mucositis condition; if human patients frequently require morphinomimetic agents to control pain, [52] yet rat pain is controlled by less potent opioids or NSAIDs, is the subjective pain experience of both these species too different to reconcile *or*, is there a possibility that partial opioid agonists, NSAIDs, or a combination thereof, are under-utilized in human oncologic practice.

In conclusion, this study has provided evidence that carprofen is efficacious against pain, and produces minimal interference with research outcomes, in a rat model of chemotherapy-induced mucositis. Furthermore, the identification of intestinal anti-inflammatory activity of μ- receptor opioid agonists supports further pre-clinical investigation into opioid agents with non-traditional pharmacological actions, such as partial agonists, atypical agents, or peripherally-acting agents [47].

## Supporting Information

S1 File Clinical and post-mortem data used in Tables 2 and 4, and 5. (XLSX)

S2 File Behavioural data used in Fig 1 and Table 3. (XLSX)

S3 File Myeloperoxidase data used in Fig 2. (XLSX)

S4 File Histological data used in Figs 4 and 5. (XLSX)

## References

[1] Sonis ST. A biological approach to mucositis. J Support Oncol. 2004; 2: 21–32. 15330370

[2] Brown CG, Wingard J. Clinical consequences of oral mucositis. Semin Oncol Nurs. 2004; 20: 16–21. 15038513 10.1053/j.soncn.2003.10.004

[3] Clarke JM, Pelton NC, Bajka BH, Howarth GS, Read LC, Butler RN. Use of the 13C-sucrose breath test to assess chemotherapy-induced small intestinal mucositis in the rat. Cancer Biol Ther. 2006;5(1): 34–8. 16294027 10.4161/cbt.5.1.2235

[4] Pico JL, Avila-Garavito A, Naccache P. Mucositis: its occurrence, consequences, and treatment in the oncology setting. Oncologist. 1998;3: 446–51. 10388137

[5] Sonis ST. Pathobiology of mucositis. Semin Oncol Nurs. 2004;20(1):11–5. 15038512 10.1053/j.soncn.2003.10.003

[6] Harris DJ. Cancer treatment-induced mucositis pain: strategies for assessment and management. Ther Clin Risk Manag. 2006;2(3): 251–8. 18360600 10.2147/tcrm.2006.2.3.251 PMC1936261

[7] Thompson WG, Longstreth GF, Drossman DA, Heaton KW, Irvine EJ, Müller-Lissner SA. Functional bowel disorders and functional abdominal pain. Gut 1999;45(suppl 2): II43–II7. 10457044 10.1136/gut.45.2008.ii43 PMC1766683

[8] Bowen JM, Gibson RJ, Keefe DMK. Animal models of mucositis: implications for therapy. J Support Oncol 2011;9: 161–8. 10.1016/j.suponc.2011.04.009 22024303

[9] Wang H, Brook CL, Whittaker AL, Lawrence A, Yazbeck R, Howarth GS. Effects of *Streptococcus thermophilus* TH-4 in a rat model of doxorubicin-induced mucositis. Scand J Gastroentero. 2013;48(8): 959–68. 10.3109/00365521.2013.812142 23865592

[10] Reinke D, Kritas S, Polychronopoulos P, Skaltsounis AL, Aligiannis N, Tran CD. Herbal substance, Acteoside, alleviates intestinal mucositis in mice. Gastroent Res Pract. 2015; 9: doi: 10.1155/2015/327872 PMC4300033 25628651

[11] Whittaker AL, Leach MC, Preston FL, Lymn KA, Howarth GS. Effects of acute chemotherapy-induced mucositis on spontaneous behaviour and the grimace scale in laboratory rats. Lab Anim. 2015; 10.1177/0023677215595554 26162377

[12] Lutfy K, Eitan S, Bryant CD, Yang YC, Saliminejad N, Walwyn W, et al Buprenorphine-induced antinociception is mediated by mu-opioid receptors and compromised by concomitant activation of opioid receptor-like receptors. J Neurosci. 2003;23(32): 10331–7. 14614092 10.1523/JNEUROSCI.23-32-10331.2003 PMC6741014

[13] Davis MP. Twelve reasons for considering buprenorphine as a frontline analgesic in the management of pain. J Support Oncol. 2012;10(6): 209–19. 10.1016/j.suponc.2012.05.002 22809652

[14] Raffa RB, Friderichs E, Reimann W, Shank RP, Codd EE, Vaught JL. Opioid and nonopioid components independently contribute to the mechanism of action of tramadol, an 'atypical' opioid analgesic. J Pharmacol Exper Ther. 1992;260(1): 275–85. 1309873

[15] Riendeau D, Charleson S, Cromlish W, Mancini JA, Wong E, Guay J. Comparison of the cyclooxygenase-1 inhibitory properties of nonsteroidal anti-inflammatory drugs and selective COX-2 inhibitors, using sensitive microsomal and platelet assays. Can J Physiol Pharmacol. 1997;75(9): 1088–95. 9365818

[16] Thomas DJ, Caffrey TC. Lipopolysaccharide induces double-stranded DNA fragmentation in mouse thymus:protective effect of zinc pretreatment. Toxicol. 1991;68: 327–37. 10.1016/0300-483x(91)90078-f 1910215

[17] Mashtoub S, Feo B, Whittaker AL, Lymn KA, Martinez-Puig D, Howarth GS. Oral Nucleotides Only Minimally Improve 5-Fluorouracil-Induced Mucositis in Rats. Nutr. Cancer. 2015; 67: 994–1000. 10.1080/01635581.2015.1062118 26284427

[18] Newcastle University. Procedures with Care. Available: http://www.procedureswithcare.org.uk/subcutaneous-injection-in-the-rat/. Accessed 7 April 2016.

[19] National Health and Medical Research Council. Australian code for the care and use of animals for scientific purposes 8th ed. Canberra: Australian Government; 2013.

[20] Hänninen L, Pastell M. CowLog: Open source software for coding behaviors from digital video. Behav Res Methods. 2009;41(2): 472–6. 10.3758/BRM.41.2.472 19363187

[21] Roughan JV, Flecknell PA. Behavioural effects of laparotomy and analgesic effects of ketoprofen and carprofen in rats. Pain. 2001;90: 65–74. 11166971 10.1016/s0304-3959(00)00387-0

[22] Miller AL, Flecknell PA, Leach MC, Roughan JV. A comparison of a manual and an automated behavioural analysis method for assessing post-operative pain in mice. Appl Anim Behav Sci. 2011;131(3–4): 138–44.

[23] Krawisz JE, Sharon P, Stenson WF. Quantitative assay for acute intestinal inflammation based on myeloperoxidase activity. Assessment of inflammation in rat and hamster models. Gastroenterology. 1984;87(6): 1344–50. 6092199

[24] Howarth GS, Francis GL, Cool JC, Xu X, Byard RW, Read LC. Milk growth factors enriched from cheese whey ameliorate intestinal damage by methotrexate when administered orally to rats. J Nutr. 1996;126(10): 2519–30. 8857513 10.1093/jn/126.10.2519

[25] Roughan JV, Flecknell PA. Evaluation of a short duration behaviour-based post-operative pain scoring system in rats. Eur J Pain. 2003;7(5): 397–406. 12935791 10.1016/S1090-3801(02)00140-4

[26] Leach MC, Klaus K, Miller AL, Scotto di Perrotolo M, Sotocinal SG, Flecknell PA. The assessment of post-vasectomy pain in mice using behaviour and the mouse grimace scale. PLoS One. 2012;7 (4):e35656 10.1371/journal.pone.0035656 22558191 PMC3338444

[27] Kalliokoski O, Jacobsen KR, Darusman HS, Henriksen T, Weimann A, Poulsen HE, et al Mice do not habituate to metabolism cage housing—a three week study of male BALB/c mice. PLoS One. 2013; 8: e58460 10.1371/journal.pone.0058460 23505511 PMC3591308

[28] Whittaker AL, Lymn KA, Howarth GS. The effects of metabolic cage housing on rat behavior, and performance in the social interaction test. J Appl AnimWelf Sci.2016; 10.1080/10888705.2016.1164048 27057787

[29] Yim GK, Lowy MT. Opioids, feeding, and anorexias. Fed Proc. 1984;43(14):2893–7. 6149154

[30] Leander JD. Evidence that nalorphine, butorphanol and oxilorphan are partial agonists at a kappa-opioid receptor. Eur J Pharmacol. 1983;86(3–4): 467–70. 6131829 10.1016/0014-2999(83)90198-x

[31] Reid LD. Endogenous opioid peptides and regulation of drinking and feeding. The Am J Clin Nutr. 1985;42(5): 1099–132. 2865892 10.1093/ajcn/42.5.1099

[32] Dayer P, Desmeules J, Collart L. Pharmacology of tramadol. Drugs. 1997;53 Suppl 2: 18–24. 9190321 10.2165/00003495-199700532-00006

[33] Whittaker AL, Howarth GS. Use of spontaneous behaviour measures to assess pain in laboratory rats and mice: How are we progressing? Appl Anim Behav Sci. 2014;151: 1–12.

[34] Richardson CA. The power of automated behavioural homecage technologies in characterizing disease progression in laboratory mice: A review. Appl Anim Behav Sci. 2015;163: 19–27.

[35] Eisenstein TK. Opioids and the immune system: what is their mechanism of action? Brit J Pharmacol. 2011;164(7): 1826–8. 21627636 10.1111/j.1476-5381.2011.01513.x PMC3246707

[36] Mauri A, Melis MR, Deiana P, Loviselli A, Volpe A, Argiolas A. Melanocortins and opioids modulate early postnatal growth in rats. Regul Pept. 1995;59(1): 59–66. 12506415 10.1016/0167-0115(95)00074-l

[37] Bryant HU, Bernton EW, Holaday JW. Immunosuppressive effects of chronic morphine treatment in mice. Life Sci. 1987;41(14): 1731–8. 3498869 10.1016/0024-3205(87)90601-1

[38] Arora PK, Fride E, Petitto J, Waggie K, Skolnick P. Morphine-induced immune alterations in vivo. Cell Immunol. 1990;126(2): 343–53. 2107031 10.1016/0008-8749(90)90326-m

[39] van der Laan JW, Krajnc EI, Krajnc-Franken MA, van Loveren H. Immunotoxicological screening of morphine and methadone in an extended 28 day study in rats. Int J Immunopharmacol. 1995;17(6): 535–43. 7499032 10.1016/0192-0561(95)00010-y

[40] Herberman RB, Ortaldo JR. Natural killer cells: their roles in defenses against disease. Science. 1981;214(4516): 24–30. 7025208 10.1126/science.7025208

[41] Zhang EY, Xiong J, Parker BL, Chen AY, Fields PE, Ma X, et al Depletion and recovery of lymphoid subsets following morphine administration. Brit J Pharmacol. 2011;164(7): 1829–44. 21557737 10.1111/j.1476-5381.2011.01475.x PMC3246708

[42] Van Loveren H, Gianotten N, Hendriksen CF, Schuurman HJ, Van der Laan JW. Assessment of immunotoxicity of buprenorphine. Lab Anim. 1994;28(4): 355–63. 7830376 10.1258/002367794780745119

[43] Sacerdote P. Opioids and the immune system. Palliat Med. 2006;20 Suppl 1: s9–15. 16764216

[44] Braun TP, Szumowski M, Levasseur PR, Grossberg AJ, Zhu XX, Agarwal A, et al Muscle atrophy in response to cytotoxic chemotherapy is dependent on intact glucocorticoid signaling in skeletal muscle. PLoS ONE. 2014;9(9): e106489 10.1371/journal.pone.0106489 25254959 PMC4177815

[45] Poonyachoti S, Portoghese PS, Brown DR. Characterization of opioid receptors modulating neurogenic contractions of circular muscle from porcine ileum and evidence that delta- and kappa-opioid receptors are coexpressed in myenteric neurons. J Pharmacol Expt Ther 2001;297(1): 69–77. 11259529

[46] Calignano A, Moncada S, Di Rosa M. Endogenous nitric oxide modulates morphine-induced constipation. Biochem Biophys Res Commun. 1991;181(2): 889–93. 1755865 10.1016/0006-291x(91)91274-g

[47] Philippe D, Dubuquoy L, Groux H, Brun V, Chuoï-Mariot MTV, Gaveriaux-Ruff C, et al Anti-inflammatory properties of the μ opioid receptor support its use in the treatment of colon inflammation. J Clin Invest 2003;111(9): 1329–38. 12727924 10.1172/JCI16750 PMC154442

[48] Kapitzke D, Vetter I, Cabot PJ. Endogenous opioid analgesia in peripheral tissues and the clinical implications for pain control. Ther Clin Risk Manag 2005;1(4): 279–97. 18360571 PMC1661636

[49] Pekoe G, Van Dyke K, Peden D, Mengoli H, English D. Antioxidation theory of non-steroidal anti-inflammatory drugs based upon the inhibition of luminol-enhanced chemiluminescence from the myeloperoxidase reaction. Agents Actions. 1982;12(3): 371–6. 6291348 10.1007/BF01965406

[50] Cata JP, Weng H-R, Dougherty PM. The effects of thalidomide and minocycline on taxol-induced hyperalgesia in rats. Brain Res. 2008;1229: 100–10. 10.1016/j.brainres.2008.07.001 18652810 PMC2577234

[51] Gibson RJ, Coller JK, Wardill HR, Hutchinson MR, Smid S, Bowen JM. Chemotherapy-induced gut toxicity and pain: involvement of TLRs. Support Care Cancer. 2015; 10.1007/s00520-015-3020-2 26581898

[52] Barasch A, Elad S, Altman A, Damato K, Epstein J. Antimicrobials, mucosal coating agents, anesthetics, analgesics, and nutritional supplements for alimentary tract mucositis. Support Care Cancer. 2006;14(6): 528–32. 16775648 10.1007/s00520-006-0066-1

